# Cutaneous Alternariosis Caused by *Alternaria infectoria*: A Case Report in Kidney Transplant Recipient and Literature Review

**DOI:** 10.3390/jof12010032

**Published:** 2025-12-31

**Authors:** Maria Antonietta Grignano, Marilena Gregorini, Tefik Islami, Maria Carmela Esposto, Camilla Vassallo, Angela Di Matteo, Elena Seminari, Palma Minutillo, Eleonora Francesca Pattonieri, Emma Diletta Stea, Giuseppe Lanotte, Valentina Portalupi, Andreana De Mauri, Elisabetta Margiotta, Alessandro Tragni, Grazia Soccio, Caterina Cavanna, Teresa Rampino

**Affiliations:** 1 Unit of Nephrology, Dialysis and Renal Transplant, Istituto di Ricovero e Cura a Carattere Scientifico Policlinico San Matteo Foundation, Piazzale Golgi 19, 27100 Pavia, Italy; ma.grignano@smatteo.pv.it (M.A.G.); e.pattonieri@smatteo.pv.it (E.F.P.); e.stea@smatteo.pv.it (E.D.S.); v.portalupi@smatteo.pv.it (V.P.); a.demauri@smatteo.pv.it (A.D.M.); el.margiotta@smatteo.pv.it (E.M.); g.soccio@smatteo.pv.it (G.S.); t.rampino@smatteo.pv.it (T.R.); 2 Department of Internal Medicine and Therapeutics, University of Pavia, Via Aselli 43/45, 27100 Pavia, Italy; giuseppe.lanotte01@universitadipavia.it (G.L.); alessandro.tragni01@universitadipavia.it (A.T.); 3 Department of Biomedical Sciences for Health, Università degli Studi di Milano, 20133 Milan, Italy; maria.esposto@unimi.it; 4 Department of Clinical-Surgical, Diagnostic and Paediatric Science, Institute of Dermatology, Istituto di Ricovero e Cura a Carattere Scientifico Policlinico San Matteo Foundation, Piazzale Golgi 19, 27100 Pavia, Italy; c.vassallo@smatteo.pv.it; 5 Clinic of Infectious Diseases, Istituto di Ricovero e Cura a Carattere Scientifico Policlinico San Matteo Foundation, Piazzale Golgi 19, 27100 Pavia, Italy; a.dimatteo@smatteo.pv.it (A.D.M.); e.seminari@smatteo.pv.it (E.S.); 6 Anatomic Pathology Unit, Istituto di Ricovero e Cura a Carattere Scientifico Policlinico San Matteo Foundation, Piazzale Golgi 19, 27100 Pavia, Italy; p.minutillo@smatteo.pv.it; 7 Microbiology and Virology Department, Istituto di Ricovero e Cura a Carattere Scientifico Policlinico San Matteo Foundation, Piazzale Golgi 19, 27100 Pavia, Italy; c.cavanna@smatteo.pv.it

**Keywords:** renal transplant, phaeohyphomycosis, *Alternaria* spp., cutaneous fungal infection, post-transplant complications

## Abstract

Cutaneous infections caused by dematiaceous fungi are rare in the general population but are increasingly recognized in solid organ transplant recipients as a consequence of prolonged immunosuppression. When Alternaria species are confirmed as the causative agents of a skin infection, the condition is referred to as alternariosis. These infections may clinically resemble bacterial or neoplastic lesions and require accurate diagnosis and individualized therapy. We report one case of cutaneous alternariosis in a kidney transplant recipient receiving tacrolimus-based immunosuppression. The patient was a 47-year-old woman who sustained minor trauma to her knee three months after transplantation. She developed an ulcerated, crusted lesion, which coincided with severe neutropenia. Histology, culture and molecular identification confirmed *A. infectoria*. Treatment included systemic azole therapy (voriconazole followed by isavuconazole) and surgical excision, resulting in resolution without recurrence. This case highlights the importance of early recognition of alternariosis in transplant recipients. Successful management typically requires combined surgical and systemic antifungal therapy, with careful monitoring of drug interactions and immunosuppressive levels to prevent toxicity or rejection.

## 1. Background

Skin lesions due to viruses or bacteria are common in transplant recipients who are on immunosuppressive therapy [1]. Skin opportunistic infections are rarely reported, but they represent a relevant clinical challenge [2,3]. Among opportunistic infections, those caused by dematiaceous fungi are noteworthy.

Phaeohyphomycosis is a histopathologic descriptor characterized by pigmented septate hyphae in tissue and does not correspond to a specific fungal genus or species. In contrast, cutaneous alternariosis is a clinical diagnosis applied when *Alternaria* species are confirmed as the causative agents of a skin infection by culture or molecular methods, most commonly involving species such as *A. alternata*, *A. infectoria*, and *A. tenuissima* [4].

*Alternaria* spp. are dematiaceous molds that produce dark gray colonies as a result of melanin-like pigment in their cell wall. These saprophytic fungi, found in soil and plants, predominantly in humid environments, can cause opportunistic infections, especially in immunocompromised patients. Skin trauma, with subsequent inoculation of fungal spores, appears to be a main route of entry [5].

Here, we describe one case of alternariosis due to *A. infectoria* occurring in renal transplant recipients. In addition, we reviewed previously reported cases in the field of transplantation and included a flowchart to illustrate the article review process.

## 2. Case Report Description

A 47-year-old woman with end-stage renal disease of unknown histological etiology underwent a kidney transplant from a donation after brain death donor, with three human leukocyte antigen mismatches and positive donor-specific antibodies for cytomegalovirus (CMV) and Epstein–Barr virus. The recipient had been on peritoneal dialysis for six months prior to transplantation and had a medical history of chronic obstructive pulmonary disease, diverticulosis of the sigmoid colon, and allergic asthma.

The patient received basiliximab (Pharma AG, Basel, Switzerland; 20 mg on day 0 and 4 post surgery) and methylprednisolone (500 mg on day 0 followed by gradual tapering regimen) as induction therapy. Maintenance immunosuppression consisted of tacrolimus (6.5 mg every 12 h), mycophenolate mofetil (MMF) (1 gr every 12 h), and methylprednisolone (16 mg/day).

Her postoperative course was uneventful, with early functional graft recovery, as evidenced by a serum creatinine level of 0.79 mg/dL (normal renge:0.55–1.02 mg/dL). at the time of hospital discharge. One month after transplantation, the patient was treated for a urinary tract infection caused by a multidrug-resistant strain of *Pseudomonas aeruginosa*, with meropenem.

She subsequently developed acute angle-closure glaucoma, initially managed with diuretics and later treated with laser peripheral iridotomy.

The patient also developed erosive gastritis associated with a reactivation of CMV infection during the post-transplant course, which required treatment with valganciclovir.

The patient resided in a rural area, frequently walking on uneven ground, and had impaired vision due to the glaucoma. These factors contributed to a fall in the field three months after transplantation, resulting in a minor laceration on her left knee, which was self-treated.

**Figure 1:**
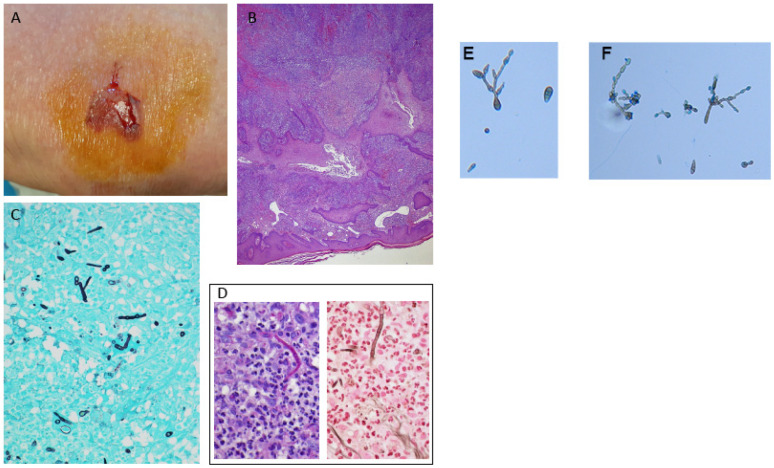
(**A**) Violaceous ulcerated lesion on the left knee. (**B**) Histological section (Hematoxylin and Eosin, original magnification ×10) showing an inflammatory infiltrate in the deep dermis. (**C**) Grocott-Gomori methenamine silver stain highlighting black fungal hyphae. (**D**) High-magnification detail (×40) of a fungal hypha stained with Periodic Acid-Schiff (PAS) and (**D**) Jones methenamine silver. Microscopic examination revealed pigmented septate hyphae (**E**) along with chains of ovate conidia possessing both transverse and vertical septa (**F**).

One week later (day 7), physical examination revealed a crusted, brownish nodule with raised red edges and a central ulcerated area (Figure 1A).

Severe neutropenia (0.57 × 10^3^/μL, normal White Blood Cells (WBC) range: 4–10 × 10^9^/L) was observed concurrently with the appearance of the lesion.

The patient was hospitalized due to suspicion of an invasive fungal infection, and both a biopsy and a cutaneous swab of the nodule were performed.

A total-body TC scan confirmed the absence of systemic involvement.

While awaiting microbiological diagnosis, antifungal therapy was initiated with voriconazole (400 mg every 12 h for the first two doses, followed by 200 mg every 12 h for 12 weeks).

Due to the onset of visual hallucinations, antifungal therapy was subsequently switched to isavuconazole (200 mg once daily), which was continued for eight weeks.

Histological analysis demonstrated necrosis and steatonecrosis involving the dermis and hypodermis, accompanied by a granulomatous inflammatory reaction characterized by a rich infiltrate of histiocytes, fibroblasts, and multinucleated giant cells (Figure 1B).

Fungal hyphae were demonstrated by Gomori–Grocott staining (Figure 1C) and were also focally highlighted by Periodic Acid–Schiff staining (PAS). They were clearly visible with Jones’ methenamine silver stain as well (Figure 1D, left and right, respectively).

We successfully isolated a positive culture from the lesions on the patient’s knee. Tissue specimens were cultured on Sabouraud dextrose agar (Merck, Darmstadt, Germany) supplemented with chloramphenicol (QUELAB, Manchester, UK) and were incubated at 30 °C for 20 days. On the ninth day of incubation, we observed some grayish colonies on the culture medium. Microscopic examination revealed pigmented septate hyphae along with chains of ovate conidia possessing both transverse and vertical septa (Figure 1E,F). This confirmed the identity of the isolate as *Alternaria* spp. following the guidelines outlined in the *Atlas of Clinical Fungi* [6].

Subsequent species identification was accomplished by amplifying and sequencing the ITS1-5,8S-ITS2 region. The consensus obtained was used for the GenBank BLAST+ 2.17.0 Our sequence showed a query cover of 100% identity with *A. infectoria* (accession number HG324079.1). The sequence originated from this case was deposited in GenBank (accession number PX632308, https://www.ncbi.nlm.nih.gov/nuccore/3125012776, accessed on 22 December 2025).

Complete surgical excision of the lesion, combined with systemic antifungal therapy, led to the resolution of the clinical presentation. Abdominal ultrasound excluded the presence of visceral nodular involvement. To date, the patient remains in good health with excellent graft function (serum creatinine level of 1.1 mg/dL) and no recurrence of alternariosis.

## 3. Bibliographic Search, Data Extraction, and Statistical Methods

A bibliographic search of similar cases published in the English language was conducted using PubMed, combining the keywords: ‘*Alternaria*,’ ‘Alternariosis,’ ‘cutaneous,’ ‘skin,’ ‘phaeohyphomycosis,’ ‘kidney,’ ‘renal,’ ‘liver,’ ‘lung,’ ‘heart,’ ‘bone marrow,’ and ‘transplantation,’ covering the period from January 1976 to May 2025.

Two investigators independently performed data extraction from the included studies. Reports without full-text availability were also reviewed when sufficient information was provided in the abstract. Collected data included year of publication, country, patient age and gender, incubation period, site and type of lesion, systemic involvement, history of trauma, identified pathogen, type of transplantation, surgical and medical treatment, duration of antifungal therapy, clinical outcome, and immunosuppressive regimen.

We used descriptive statistics, including frequency counts, means, and medians, to characterize the pooled sample.

## 4. Discussion

In recent years, an increasing prevalence of phaeohyphomycosis has been observed among solid organ transplant recipients [2].

While rare, this infection carries a severe prognosis if not adequately treated. However, defining its true incidence remains complex, as the majority of data derive from heterogeneous case reports that often lack molecular characterization. Our case fits precisely into this context, underscoring the critical importance of molecular investigation in clinical management. Traditional diagnostic methods demonstrate evident limitations: while histological examination permits a generic diagnosis of “phaeohyphomycosis,” it fails to provide species-level identification—a factor that is a significant determinant of therapeutic success. In parallel, conventional culture often proves inconclusive; *A. infectoria*, for example, frequently presents as sterile mycelium lacking characteristic sporulation, a morphological feature that exposes the patient to a substantial risk of misdiagnosis or therapeutic delay [3,5].

Consequently, reliance on molecular identification is not merely an academic exercise but a clinical necessity, particularly in light of species-specific resistance profiles. *A. infectoria* may exhibit higher minimum inhibitory concentrations (MICs) for echinocandins and variable susceptibility to amphotericin B compared to *A. alternata* [7].

Furthermore, recent evidence indicates an alarming epidemiological shift: *A. infectoria* is emerging as the predominant species within the kidney transplant cohort, often manifesting with a more insidious and paucisymptomatic clinical course [5].

This, combined with its resistance profile, favors chronicization. These data reinforce the hypothesis that this species possesses a selective tropism and specific adaptation mechanisms for the immunosuppressed host. Therefore, the absence of molecular specification represents a major critical issue, exposing patients to ineffective empiric regimens and to treatment resistance that is only apparent—resulting, in fact, from an erroneous identification of the target pathogen.

Although molecular identification is now regarded as the gold standard for guiding therapy, its use in routine clinical practice remains insufficiently documented in the literature. Many published cases are limited to a generic diagnosis of “phaeohyphomycosis” due to technical constraints, high costs, lack of specific mycological expertise in non-referral centers, difficulties in DNA extraction from formalin-fixed tissues, or limited access to historical archival material.

This deficiency often necessitates the use of the umbrella term “phaeohyphomycosis” to describe a clinical problem that is clearly present and warrants reporting. Given these methodological considerations, our literature review identified 124 reported cases of cutaneous phaeohyphomycosis in transplant recipients. To ensure taxonomic accuracy and provide reliable data for therapeutic decision-making, we extracted and analyzed separately those cases in which alternariosis was confirmed by molecular identification (only 32 out of 124 cases), which are presented in detail in Table 1. This distinction is essential: while phaeohyphomycosis includes infections by any dematiaceous fungus, alternariosis implies unique biological characteristics and susceptibility profiles that only molecular confirmation can determine—data unfortunately lacking in most historical reports from Southwestern Europe (Supplementary File S1) [8,9,10,11,12,13,14,15,16,17,18,19,20,21,22,23,24,25,26,27,28,29,30,31,32,33,34,35,36,37,38,39,40,41,42,43,44,45,46,47,48,49,50,51,52,53,54,55,56,57,58,59,60,61,62,63,64,65,66,67,68,69,70,71,72,73,74,75,76,77,78,79,80,81,82,83,84,85,86,87,88,89,90,91,92,93,94,95,96,97,98,99,100,101,102,103].

Analyzing the clinical and epidemiological profile emerging from this review, alternariosis appears predominantly in male kidney transplant recipients—the most frequently transplanted organ worldwide—who require particularly intensive, lifelong immunosuppressive management. Our analysis confirms that the triple immunosuppressive regimen based on tacrolimus, MMF, and corticosteroids—the same regimen administered to our patient—is frequently reported in the literature as a risk factor for phaeohyphomycosis not only in transplant recipients but also in patients with other conditions, such as autoimmune diseases or diabetes [54,104,105].

The combined use of these potent immunosuppressive agents is directly associated with an increased incidence of opportunistic fungal infections in this population. Consistent with our case, the initial clinical presentation is described in most reports as a solitary lesion, typically localized to the upper or lower extremities, with a predilection for skin overlying bony prominences (e.g., the knees). This distribution is not random but often reflects occupational or recreational exposure; subcutaneous fungal infections are frequently associated with agricultural or forestry activities, contexts characterized by a male predominance and a high risk of inoculation via penetrating trauma from thorns, splinters, or nails [4].

In this scenario, skin fragility induced by chronic steroid therapy likely acts as a relevant cofactor, facilitating pathogen entry even following minor trauma [106].

This strong association with environmental exposure is supported by the ecology of the fungus itself. *Alternaria* spp. are ubiquitous but particularly prevalent in the Mediterranean region, where they thrive both in domestic environments (such as damp walls) and in nature on decaying vegetable substrates.

Their remarkable capacity for sporulation facilitates aerial dispersion and environmental persistence, consolidating their role as emerging pathogens [107,108].

Once the pathogen penetrates the host, its virulence is sustained by specific factors. Although the production of mycotoxins (tenuazonic acid, altertoxin, alternariols) is classically associated with the induction of allergic respiratory pathologies [109], the melanin-like pigment present in the cell wall plays a crucial role in invasive cutaneous infections. This pigment acts as a protective shield, allowing the fungus to neutralize free radicals and hypochlorite generated by the immune response (phagocytes and neutrophils) [110,111].

In the context of post-transplant immunosuppression, the host-*Alternaria* interaction results in a highly variable incubation period, with a median of 12 months (interquartile range: 4 months–3 years) [38,39,42,49,51,57,59,60,67,68,69,70,71,72,73,74,77,79,84,85,89,91,92,94,96,98,101,102] (Table 1 and Table 2).

However, the ubiquity of *Alternaria* spp. presents a significant diagnostic challenge: the fungus can also be isolated from healthy skin or appear as a common laboratory contaminant [107,112,113].

Therefore, culture isolation alone is insufficient to diagnose an active infection. To confirm the fungus’s etiological role, a combined approach demonstrating tissue invasion is essential—specifically, visualizing hyphae in a biopsy in association with colony identification. In this context, the absence of serological or antigenic tests for *Alternaria* spp., together with the inherent limitations of culture morphology, makes the need for the previously discussed molecular accuracy even more urgent.

It remains the only method to distinguish with certainty between pathogenic species and environmental contaminants or commensals [114].

**Figure 2:**
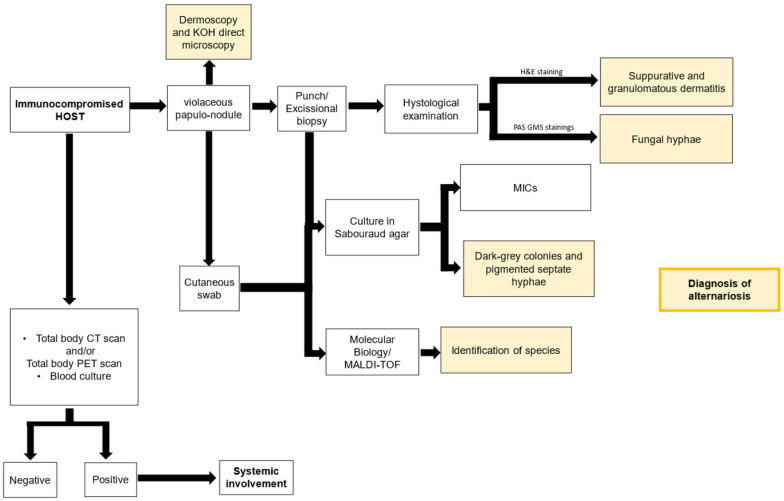
Diagnostic algorithm to be applied in suspicion of alternariosis. CT: Computed Tomography scan; GMS: Gomori–Grocott’s silver; H&E: Hematoxylin and eosin; MICs: minimum inhibitory concentrations; PET: Positron Emission Tomography. The algorithm presented is not a universally accepted standard; instead, it reflects the diagnostic pathway used in this case, informed by our institutional experience and clinical judgment.

The flow chart in Figure 2 summarizes a diagnostic algorithm to apply in suspicion of alternariosis.

On a strictly clinical level, the appearance of the lesions necessitates a rigorous differential diagnosis. The morphological presentation can mimic both infectious diseases (including chromoblastomycosis, sporotrichosis, blastomycosis, coccidioidomycosis, paracoccidioidomycosis, and cutaneous leishmaniasis) and neoplastic conditions common in transplant patients, such as squamous cell carcinoma, Kaposi’s sarcoma, or other atypical nodular lesions [114].

Given the patient’s immunosuppressed state, although the lesion in our case was solitary, and systemic involvement was instead excluded through blood cultures and total-body CT imaging.

Our systematic review confirms the rarity of such occurrences: dissemination was documented in only 8 out of 124 patients (Supplementary File S1), involving recipients of various transplant types (kidney, heart, bone marrow).

Although infrequent, the potential risk of systemic dissemination underscores the need for a thorough diagnostic work-up to promptly select appropriate therapy.

In this context, we acknowledge that serum β-D-glucan is generally considered a useful serologic adjunct in the evaluation of fungal infections and that elevated levels have been reported in cases of alternariosis. However, this test was not performed in our patient [115].

Managing these infections remains challenging due to the complete absence of randomized clinical trials. Consequently, medical decisions rely primarily on expert opinion and case series. The literature highlights important criticalities: although itraconazole has historically been the most widely used antifungal, reports of therapeutic failure or relapse are not uncommon (Supplementary File S1).

Cure rates appear decidedly more consistent when systemic therapy (including amphotericin B or new-generation azoles) is combined with local intervention (radical surgical excision or cryotherapy), suggesting the necessity of a multimodal approach.

Outcome data confirm the need for diagnostic precision. A critical vulnerability, as outlined in Supplementary File S1, is the absence of molecular typing: empirical regimens are often suboptimal, while antifungal selection based on species data and specific MICs leads to significantly superior survival and cure rates. The European Fungal Infection Study Group specifically recommends that immunocompromised patients be treated with agents such as voriconazole, posaconazole, or amphotericin B rather than less effective therapies [116,117].

Applying these principles, our patient achieved complete clinical resolution thanks to the synergistic combination of systemic voriconazole and radical surgical excision.

However, therapeutic success necessitates complex pharmacological management. Although cutaneous infections generally carry a favorable prognosis, prolonged antifungal therapy is frequently required to prevent systemic dissemination and treat potential subclinical foci—though the optimal duration remains undefined. Such prolonged treatment introduces a critical challenge in transplant patients: significant pharmacological interactions between azoles and immunosuppressants. Continuous monitoring of both agents is essential as they compete for metabolism by the CYP3A4/5 cytochrome [118], creating a precarious balance.

Moreover, unlike lung or liver transplant recipients, renal transplant patients are not routinely given universal prophylaxis against invasive fungal infections due to the low overall incidence. In this contest, standard protocols rely on trimethoprim/sulfamethoxazole for *Pneumocystis jirovecii*—with doses carefully adjusted according to glomerular filtration rate—and topical nystatin for mucocutaneous candidiasis, while systemic fluconazole is reserved for clearly defined high-risk scenarios [119].

For these reasons, therapeutic drug monitoring becomes mandatory not only to prevent overdose toxicity but also to minimize the risk of acute organ rejection.

The present study must be interpreted in light of some intrinsic limitations. First, the retrospective nature of the review and the reliance on case reports entail an inevitable publication bias, which tends to overrepresent the most severe or atypical clinical presentations to the detriment of milder forms. Second, the substantial limitation linked to diagnostic heterogeneity persists: as widely discussed, the majority of historical cases are based on purely morphological identification. This lack of molecular data in the previous literature reduces the possibility of statistically correlating individual species (and relative susceptibility profiles, e.g., *A. infectoria* vs. *A. alternata*) with long-term clinical outcomes. To bridge these knowledge gaps, a paradigm shift is necessary towards the creation of prospective multicenter registries, the only tools capable of defining the true incidence and risk factors of such a rare pathology. In parallel, future research must concentrate on the development and validation of rapid diagnostic tools, such as point-of-care molecular assays, which allow for overcoming the limits of traditional culture and promptly guiding therapeutic choices, thus improving the prognosis of transplant patients.

## 5. Conclusions

Cutaneous alternariosis is a rare but significant complication in kidney transplant recipients. Our case, combined with a comprehensive literature review, underscores the importance of a high index of suspicion, especially for nodular lesions on exposed limbs.

A definitive diagnosis requires a multimodal approach including histology, mycological culture and molecular identification. Successful management relies on a combined strategy of surgical excision and prolonged systemic antifungal therapy, with careful monitoring of drug interactions with immunosuppressants. Further studies are needed to establish standardized therapeutic guidelines.

## Figures and Tables

**Table 1:** List of 32 alternariosis cases confirmed by molecular/HPLC methods. Amph B: Amphotericin B, BMT: Bone Marrow Transplant, Cas: Caspofungin, CoT: Composite Transplant, Cryo: Cryotherapy, CyA: Cyclosporine, DDKT: Deceased Donor Kidney Transplant, DOD: Deceased for Other Disease, FK: Tacrolimus, Fluco: Fluconazole, F: Female, His: Histological examination, IG: Incubation Gap, Isa: Isavuconazole, ITS: ITS sequencing, Itra: Itraconazole, HT: Heart Transplant, KT: Kidney Transplant, LiT: Liver Transplant, LuT: Lung Transplant, M: Male, MMF: Mycophenolate Mofetil, Myc: Mycological culture, NA: Not Available, Posa: Posaconazole, RT-PCR: Real Time PCR assay, Terb: Terbinafine, Vori: Voriconazole.

Ref.	Authors	Year	Country	Gender, Age	IG (Months)	Site and Type of Lesion	Systemic Involvement	Fungal Identification Tecnique	Pathogen	Type of Transplant	Surgical Treatment	Medical Treatment	AMT Lenght (Months)	Outcome	IST
[38]	Mayser et al.	2004	Netherlands	F, 68	8	Knee, papulo-nodule	No	His, Myc, ITS	*A. alternata*	DDKT	Yes	Itra	1	Cured	FK, steroids
[39]	Pereiro et al.	2004	Netherlands	M, 66	6	Left foot, ulcerated nodule	No	His, Myc, ITS	*A. alternata*	LiT	Yes	No	0	Cured	FK, steroids
[42]	Lo Cascio et al.	2004	Italy	M, 49	10	Right arm and left leg, papulo-nodule	No	His, Myc, ITS	*A. infectoria*	HT	No	Itra/Amph B	1.5	Cured	NA
[49]	Nulens et al.	2006	Netherlands	M, 64	3	Right hand, papulo-nodule	No	His, ITS	*A. infectoria*	DDKT	Yes	No	0	Cured	FK, MMF, steroids
[51]	Ara et al.	2006	Spain	M, 58	12	Legs, papulo-nodule	No	His, Myc, ITS	*Alternaria* spp.	DDKT	Yes	Itra, Cryo, Terb, Fluco	8	Cured	FK, MMF, steroids
[57]	Brasch et al.	2008	Netherlands	M, 68	NA	Left feet, papulo-nodule	No	His, Myc, ITS	*A. infectoria*	DDKT	No	Itra	2	Cured	FK, MMF, steroids
[59]	Segner et al.	2009	Belgium	M, 73	24	Right hand, plaque	No	His, Myc, RT-PCR, ITS	*A. infectoria*	DDKT	Yes	Itra	12	Cured	FK, MMF, steroids
[60]	Larsen et al.	2009	Denmark	M, 43	6	Legs, papulo-nodule	No	His, Myc, ITS	*A. alternata*	DDKT	No	Vori	5	Cured	FK, MMF, steroids
[67]	Cunha et al.	2012	Portugal	M, 53	16	Forearm, hands and tibia, papulo-nodule	No	His, Myc, ITS	*A. infectoria*	DDKT	No	Itra	10	Cured	FK, MMF, steroids
[68]	Seyfarth et al.	2012	Germany	M, 65	4	Left hand, plaque	No	His, Myc, ITS	*A. infectoria*	DDKT	No	Vori/Cas	3	Cured	FK, MMF, steroids
[69]	Tambasco et al.	2012	Italy	F, 64	NA	Legs, plaque	No	His, Myc, ITS	*Alternaria* spp.	DDKT	No	Terb	7	Cured	NA
[70]	Robert et al.	2012	France	M, 54	8	Legs, papulo-nodule	No	His, Myc, ITS	*A. infectoria*	DDKT	No	Fluco	1	Cured	FK, MMF, steroids
[70]	Robert et al.	2012	France	F, 73	60	Right hand, papulo-nodule	Yes	His, Myc, ITS	*A. infectoria*	DDKT	No	Vori	4	Cured-DOD	FK, steroids
[70]	Robert et al.	2012	France	M, 56	3	Knee, papulo-nodule	No	His, Myc, ITS	*A. infectoria*	CoT	Yes	Vori/Posa/Cas	1	Cured	CyA, MMF, steroids
[71]	Rammaert et al.	2012	France	F, 64	30	Right foot, ulcerated nodule	Yes	His, Myc, ITS	*A. infectoria*	HT	No	Itra/Posa	12	Cured	FK, MMF, steroids
[72]	Lavergne et al.	2012	France	M, 63	18	Right elbow, papulo-nodule	No	Myc, molecular identification	*A. alternata*	HT	No	Vori	10	Cured	FK, MMF, steroids
[73]	Saegeman et al.	2012	Belgium	F, 52	55	Legs, papulo-nodule	No	His, Myc, ITS	*A. infectoria*	LuT	Yes	Vori	6	DOD	FK, Aza, steroids
[74]	Severo et al.	2012	Brazil	M, 27	0,5	Legs and foot, papulo-nodule	No	His, Myc, ITS	*A. alternata*	DDKT	NA	NA	NA	NA	FK
[77]	Lopes et al.	2013	Portugal	M, 61	6	Legs, papulo-nodule	No	His, Myc, ITS	*A. infectoria*	DDKT	No	Itra	3	Cured	FK, steroids
[77]	Lopes et al.	2013	Portugal	M, 63	14	Legs, papulo-nodule	No	His, Myc, ITS	*A. infectoria*	DDKT	No	Cryo/Posa	1	Cured	CyA, steroids
[77]	Lopes et al.	2013	Portugal	M, 56	120	Right hand, papulo-nodule	No	His, ITS	*A. infectoria*	DDKT	Yes	Itra	3	Cured	FK, MMF, steroids
[79]	Secnikova et al.	2014	Czech Republic	M, 60	9	Left arm, ulcerated nodule	Yes	His, Myc, ITS	*A. alternata*	HT	Yes	Vori/Posa	7	Cured	FK, MMF, steroids
[84]	Daglar et al.	2014	Turkey	M, 33	84	Right arm and legs, papulo-nodule	No	His, RT-PCR	*A. infectoria*	DDKT	No	Itra/Amph B	3	Cured	FK, MMF, steroids
[85]	Gonzalez-Vela et al.	2014	Spain	M, 60	4	Left hand and legs, papulo-nodule	No	His, Myc, ITS	*A. triticina*	LuT	No	Itra	6	Relapse	FK, MMF, steroids
[89]	Bras et al.	2015	Portugal	M, 65	2	Legs and ketf hand, papulo-nodule	No	His, Myc, ITS	*A. alternata, A. infectoria*	LiT	Yes	Itra	3	Cured	FK, MMF, steroids
[91]	Karatas et al.	2016	Turkey	M, 48	4	Arm, papulo-nodule	No	His, Myc, ITS	*A. alternata*	DDKT	Yes	Itra	12	Cured	FK, MMF, steroids
[92]	Lyscova et al.	2017	Czech Republic	M, 61	12	Helbow, papulo-nodule	Yes	His, Myc, ITS, partial β-tubulin gene	*A. infectoria*	HT	Yes	Vori/Posa	7	Cured	FK, MMF, steroids
[94]	Liu et al.	2017	USA	M, 66	1.5	Right thumb, papulo-nodule	No	His, Myc, ITS	*A. rosae*	BMT	No	Vori/Posa	2	DOD	CyA, Sirolimus, MMF, steroids
[96]	Schuermans et al.	2017	Belgium	F, 52	NA	Forearm, papulo-nodule	No	His, ITS	*A. infectoria*	LDKT	Yes	Vori/Itra	NA	Cured	FK, MMF, steroids
[98]	Dalla Gasperina et al.	2019	Italy	M, 68	58	Left hand, plaque	No	His, Myc, ITS	*A. alternata*	KT	No	Vori/Isa/Posa	1	Cured	FK, MMF, steroids
[101]	Campoli et al.	2020	Italy	F, 56	2	Diffuse ulcerated papules	No	His, Myc, MALDI-TOF MS	*A. alternata*	LiT	Yes	Vori	6	Cured	NA
[102]	Maisons	2022	France	M, 69	48	Knee, ulcerated nodule	No	His, Myc, LSU	*A. infectoria*	HT	Yes	Isa/Terb	12	Cured	FK, MMF, steroids

**Table 2:** Treatments and outcomes of the 32 cases of cutaneous alternariosis confirmed by molecular/HPLC methods in transplanted patients.

Therapeutic Approach	No. of Patients and (%)
Antifungal drugs alone	15 (46.9)
Surgical treatment alone	2 (6.2)
Thermotherapy alone	0 (0.0)
Combined treatments	
Surgery and Drugs	12 (37.5)
Cryotherapy and Drugs	1 (3.1)
Surgery, Cryotherapy and Drugs	1 (3.1)
Local treatment	0 (0.0)
NO treatment	0 (0.0)
Unavailable data	1 (3.1)
Pharmacological therapy *	**No. of patients and (%)**
Itroconazole	14 (43.7)
Amphotericin B	2 (6.2)
Voriconazole	12 (37.5)
Terbinafine	3 (9.4)
Fluconazole	2 (6.2)
Posaconazole	7 (21.9)
Ketoconazole	0 (0.0)
Caspofungin	2 (6.2)
Flucitosine	0 (0.0)
	**Duration of drug therapy** **(Months)** **Median (CI)**
	3.5 (1.4–7.2)
**Outcomes**	**No. of patients and (%)**
Cured	27 (84.4)
Died of Other Diseases	3 (9.4)
Relapse	1 (3.1)
Lost at follow-up	0 (0.0)
Unavailable data	1 (3.1)
Deceased for Alternariosis	0 (0.0)

* Many patients were treated with poly-therapy. No., Number; CI, Confidence Interval.

## Data Availability

The data presented in this study are openly available in GenBank under accession number PX632308, https://www.ncbi.nlm.nih.gov/nuccore/3125012776, accessed on 22 December 2025.

## Supplementary Materials

The following supporting information can be downloaded at: https://www.mdpi.com/article/10.3390/jof12010032/s1, File S1: *Alternaria* phaeohyphomycosis: Clinical and Microbiological data from literature review. Description: All patient collected data have been summarized in table format. Abbreviations: AMT: Average of Medical Therapy, Aza: Azathioprine, Amph B: Amphotericin B, Cas: Caspofungin, Clot: Clotrimazole, CoT: Composite Transplant, CPX: Ciclopiroxolamine, Cryo: Cryotherapy, CyA: Cyclosporine, DDKT: Deceased Donor Kidney Transplant, DOD: Died of Other Diseases, Fluco: Fluconazole, F: Female, FK: Tacrolimus, Eco: Econazole, HT: Heart Transplant, His: Histological examination, IG: Incubation Gap, Isa: Isavuconazole. Itra: Itraconazole, ITS: ITS sequencing, Keto: Ketoconazole, LSU: LSU sequencing, LDKT: Living Donor Kidney Transplant, LiT: Liver Transplant, LuT: Lung Transplant, M: Male, MMF: Mycophenolate Mofetil, Myc: Mycological culture, NA: Not Available, Posa: Posaconazole, PT: Pancreas Transplant, RT-PCR: Real Time PCR assay, Terb: Terbinafine, Thermo: Thermotherapy, Vori: Voriconazole.

## Abbreviations

The following abbreviations are used in this manuscript:

AMT	Average of Medical Therapy
Amph B	Amphotericin B
Aza	Azathioprine
Cas	Caspofungin
Clot	Clotrimazole
CMV	Cytomegalovirus
CoT	Composite Transplant
CPX	Ciclopiroxolamine
Cryo	Cryotherapy
CyA	Cyclosporine
DDKT	Deceased Donor Kidney Transplant
DOD	Died of Other Diseases
Eco	Econazole
Fluco	Fluconazole
FK	Tacrolimus
HT	Heart Transplant
His	Histological examination
IG	Incubation Gap
Isa	Isavuconazole
Itra	Itraconazole
ITS	ITS sequencing
Keto	Ketoconazole
LSU	LSU sequencing
LDKT	Living Donor Kidney Transplant
LiT	Liver Transplant
LuT	Lung Transplant
M	Male
MIC	minimum inhibitory concentrations
MMF	Mycophenolate Mofetil
Myc	Mycological culture
NA	Not Available
PAS	Periodic acid-Schiff staining
Posa	Posaconazole
PT	Pancreas Transplant
RT-PCR	Real Time PCR assay
Terb	Terbinafine
Thermo	Thermotherapy
Vori	Voriconazole
WBC	White Blood Cells
