# Supplementary material for: Cutaneous Alternariosis Caused by Alternaria infectoria: A Case Report in Kidney Transplant Recipient and Literature Review
Source: J Fungi (Basel). 2025 Dec 31;12(1):32. doi: 10.3390/jof12010032 (PMC12843189; doi:10.3390/jof12010032)
Supplement: Supplementary file 1 [file jof-12-00032-s001.zip › jof-3896918-supplementary.pdf]

Ref.	Authors	Year	Country	Gender, Age	IG (months)	Site and type of lesion	Systemic involvement	Fungal identification tecnique	Pathogen	Type of transplant	Surgical treatment	Medical treatment	AMT lenght (months)	Outcome	IST
8	Pedersen et al	1976	Sverige	M,63	7	Hand, ulcerated nodule	No	His	*Alternaria* spp	DDKT	No	Local treatment	11	DOD	Aza, steroids
9	Chevrant-Breton et al	1981	France	F,38	15	Knee and legs, pustules	No	NA	*Alternaria* spp	DDKT	No	Eco	NA	Cured	Aza, steroids
9	Chevrant-Breton et al	1981	France	M,35	38	Hand, ulcerated nodule	No	NA	*Alternaria* spp	DDKT	Yes	Keto	2	Cured	Aza
10	Bourlond et al	1984	Belgium	M,32	NA	Legs, papulo-nodule	No	His , Myc	*A.alternata*	DDKT	No	Keto	NA	Cured	Aza, steroids
11	Blanc et al	1984	France	M,46	NA	Legs and foot, ulcerated nodule	NA	NA	*A.chartarum*	DDKT	Yes	No	NA	Cured	NA
12	Laudren et al	1985	France	M,53	72	Forehead and hand, plaque	No	NA	*A.tenuissima*	DDKT	No	No	0	Stable	NA
13	Aloi et al	1987	Italy	F,51	24	Hand, papulo-nodule	No	NA	*A.alternata*	DDKT	No	Keto	2	Cured	CyA, Aza
14	Drouhet et al	1991	France	M,40	NA	Knee, pustules	No	NA	*A.alternata*	DDKT	No	No	NA	NA	Aza, steroids
15	Repiso et al	1993	Spain	NA,48	11	Legs, papulo-nodule	No	His , Myc	*A.alternata*	LiT	No	Itra	4	Cured	Aza, steroids
16	Shearer et al	1993	USA	M,13	0,25	Knee, papulo-nodule	No	NA	*Alternaria* spp	BMT	Yes	Amph B	1	Cured	CyA, steroids
17	Becherel et al	1995	France	F,50	12	Legs, papulo-nodule	No	His , Myc	*Alternaria* spp	DDKT	No	Local treatment	3	Cured	Aza, steroids
18	Romano et al	1997	Italy	M,55	NA	Legs, papulo-nodule	No	His , Myc	*A.tenuissima*	DDKT	Yes	Itra	2	Cured	CyA, steroids
19	Viera Mota et al	1998	Portugal	F,56	NA	Legs, papulo-nodule	NA	NA	*A.tenuissima*	DDKT	No	Itra	NA	NA	NA
20	Acland et al	1998	UK	M,70	2	Legs, ulcerated nodule	No	His , Myc	*A.alternata*	DDKT	No	Itra	2,5	Cured	CyA, Aza, steroids
21	Laumaille et al	1998	France	M,58	NA	NA	NA	His	*A.infectoria*	LiT	Yes	No	NA	Cured	NA
22	Bartolome et al	1999	Spain	M,33	3	Legs and right arm, papulo-nodule	No	His , Myc	*A.chlamydospora*	BMT	No	Amph B	1	Cured	NA
23	Magina et al	2000	Portugal	F,63	11	Nasal septum, legs and knee, papulo-nodule	No	His, TEM , Myc	*A.chartarum*	DDKT	No	Itra	3	Cured	Aza, steroids
24	Altomare et al	2000	Italy	F,58	39	Legs, papulo-nodule	No	His , Myc	*A.tenuissima*	DDKT	Yes	Terb	2	Cured	CyA, Aza, steroids
25	Baykal et al	2000	Turkey	F,52	60	Right hand, plaque	No	His , Myc	*Alternaria* spp	DDKT	No	Itra	1	DOD	NA
26	Romero et al	2000	USA	M,44	4	Left foot, papulo-nodule	No	His , Myc	*Alternaria* spp	DDKT	Yes	Itra/ Amph B	3,5	Cured	NA
27	Vieira Mota et al.	2001	Portugal	F,56	12	Left leg, papulo-nodule	No	His , Myc	*A.tenuissima*	KT	No	Itra	NA	NA	CyA, steroids
28	Benito et al	2001	Spain	M,63	3	Arms and legs, papulo-nodule	No	His , Myc	*A.alternata*	LiT	No	Itra/ Amph B	6	Cured	FK, steroids
29	Halaby et al	2001	Belgium	M,60	5	Right thumb and feet, papulo-nodule	Yes	His , Myc	*A.infectoria*	DDKT	Yes	Itra/ Amph B	2	Cured	FK, steroids
30	Gilmour et al	2001	UK	M,53	9	Legs and arms, papulo-nodule	No	His , Myc	*A.alternata*	HT	Yes	Fluco/ Itra/ Cryo	2	Cured	FK, steroids
31	Gerdsen et al	2001	Germany	F,60	60	Left hand and left Elbow, papulo-nodule	No	NA	*A.infectoria*	HT	No	Itra	4	Cured	FK, MMF, steroids
32	Miele et al	2002	USA	M,59	7	Legs, papulo-nodule	No	His , Myc	*Alternaria* spp	HT	No	Itra	2	Cured	FK, MMF, steroids
33	Eguino et al	2002	Spain	F,67	11	Legs, papulo-nodule	No	NA	*Alternaria* spp	DDKT	Yes	Amph B	0,5	Cured	FK, MMF, steroids
34	Diz et al	2003	Spain	M,51	9	Forearm, papulo-nodule	No	NA	*A.alternata*	DDKT	Yes	Itra	12	Cured	FK, MMF, steroids
35	Merino et al	2003	Spain	M,58	48	Forearms, papulo-nodule	No	His , Myc	*Alternaria* spp	DDKT	No	Itra/ Amph B	10	Cured	CyA, Aza, steroids
36	Kim et al	2003	Korea	M,55	4	Legs, papulo-nodule	No	His , Myc	*Alternaria* spp	DDKT	Yes	No	0	Cured	FK, MMF, steroids
37	Noack-Wlemers et al	2003	Netherlands	M,60	NA	Feet and thumb, ulcerated nodule	NA	His, Myc , serologic testing	*A.alternata*	DDKT	No	Itra/ Amph B	NA	Cured	NA
38	Mayser et al	2004	Netherlands	F,68	8	Knee, papulo-nodule	No	His, Myc , ITS	*A. alternata*	DDKT	Yes	Itra	1	Cured	FK, steroids
39	Pereiro et al	2004	Netherlands	M,66	6	Left foot, ulcerated nodule	No	His, Myc , ITS	*A. alternata*	LiT	Yes	No	0	Cured	FK, steroids
40	Kazory et al	2004	France	M,58	6	Legs, papulo-nodule	No	His , Myc	*Alternaria* spp	DDKT	Yes	Itra	3	Cured	CyA, Aza, steroids
40	Kazory et al	2004	France	M,41	6	Knee, papulo-nodule	No	His, Myc	*A.alternata*	DDKT	No	Itra	3	Relapse	FK, Aza, steroids
41	Yehia et al	2004	New Zealand	M,65	4	Legs, ulcerated nodule	No	His, Myc	*A.alternata*	DDKT	No	Terb/ Itra	6	Cured	FK, MMF
41	Yehia et al	2004	New Zealand	F,54	8	Legs, papulo-nodule	No	His, Myc	*A.alternata*	LDKT	Yes	Itra	NA	Cured	FK, MMF
42	Lo Cascio et al	2004	Italy	M,49	10	Right arm and left leg, papulo-nodule	No	His, Myc , ITS	*A. infectoria*	HT	No	Itra/ Amph B	1,5	Cured	NA
43	Ortiz et al	2004	Spain	F,45	NA	Legs and elbows, papulo-nodule	Yes	His , Myc	*A.alternata*	BMT	No	Itra/ Amph B	NA	DOD	NA
44	Gilaberte et al	2005	Spain	M,27	24	Legs, thigh and arm, papulo-nodule	No	His , Myc	*A.infectoria*	LuT	Yes	Amph B + Fluo/ Terb	7	Cured	FK, steroids
44	Gilaberte et al	2005	Spain	M,48	11	Legs, papulo-nodule	No	His , Myc	*A.alternata*	LiT	No	Itra	4	Cured	FK, steroids
44	Gilaberte et al	2005	Spain	M,66	24	Legs, papulo-nodule	No	His , Myc	*A.infectoria*	HT	No	Itra/ Terb	11	Cured	CyA, Aza, steroids
44	Gilaberte et al	2005	Spain	M,65	48	Legs, ulcerated nodule	No	His , Myc	*Alternaria* spp	DDKT	No	Amph B	7	DOD	Aza, steroids
44	Gilaberte et al	2005	Spain	M,67	24	Right thigh, plaque	No	His , Myc	*Alternaria* spp	DDKT	No	Fluco	7	Lost	CyA, steroids
44	Gilaberte et al	2005	Spain	M,63	2	Legs, papulo-nodule	No	His , Myc	*Alternaria* spp	HT	No	Fluco	5	Cured	CyA, Aza, steroids
44	Gilaberte et al	2005	Spain	M,53	NA	Left gluteus, plaque	No	His , Myc	*Alternaria* spp	DDKT	No	Itra	3	Lost	NA
44	Gilaberte et al	2005	Spain	M,55	6	Knee, plaque	No	His , Myc	*A.alternata*	DDKT	No	Thermo	0	Cured	FK, MMF, steroids
44	Gilaberte et al	2005	Spain	F,67	132	Foot, plaque	No	His , Myc	*Alternaria* spp	LiT	No	Terb/ Itra	4	Cured	FK, steroids
45	Torres-Rodriguez et al	2005	Spain	M,55	2	Knee, plaque	No	His , Myc	*A.alternata*	DDKT	No	Thermo	12	Cured	FK, MMF, steroids
46	Romano et al	2005	Italy	M,52	1	Right forearm, papulo-nodule	No	His , Myc	*A.alternata*	DDKT	No	Itra	1	NA	CyA, steroids
47	Robertshaw et al	2005	UK	M,28	6,5	Knee, papulo-nodule	No	His , Myc	*A.Tenuissima*	DDKT	Yes	Itra	1	Cured	FK, Aza, steroids
48	Vieira et al	2006	Portugal	M,67	8	Forearm and knee, papulo-nodule	No	His , Myc	*A.infectoria*	LiT	No	No	0	Cured	FK, MMF, steroids
49	Nulens et al	2006	Netherlands	M,64	3	Right hand, papulo-nodule	No	His , ITS	*A. infectoria*	DDKT	Yes	No	0	Cured	FK, MMF, steroids
50	Gallelli et al	2006	Italy	M,60	36	Right forearm and legs, ulcerated nodule	No	His , Myc	*A.infectoria*	DDKT	No	Itra	5	Cured	FK, MMF, steroids
51	Ara et al	2006	Spain	M,58	12	Legs, papulo-nodule	No	His, Myc , ITS	*Alternaria* spp	DDKT	Yes	Itra/ Cryo/ Terb / Fluco	8	Cured	FK, MMF, steroids
52	Henn et al	2006	USA	F,47	0,3	Hand and Feet, papulo-nodule	Yes	His	*Alternaria* spp	BMT	No	Vori/Amph B	NA	Cured	NA
53	Luque et al	2006	Spain	M,62	3	Knee, ulcerated nodule	No	Myc	*A.alternata*	LiT	Yes	Vori/Amph B	1	Cured	CyA, steroids
54	Farina et	2007	Italy	M,61	96	Knee, ulcerated nodule	No	His , Myc	*A.alternata*	DDKT	No	Itra	1	DOD	CyA, steroids
55	Singh et al	2007	UK	F,47	3	Left ankle, papulo-nodule	No	His , Myc	*A.alternata*	DDKT	No	Vori/ Itra	5	Cured	FK, steroids
56	Garduno et al	2007	Spain	M,64	8	Hand, plaque	No	NA	*A.infectoria*	DDKT	Yes	Itra	9	Cured	FK, MMF, steroids
57	Brasch et al	2008	Netherlands	M,68	NA	Left feet, papulo-nodule	No	His, Myc , ITS	*A. infectoria*	DDKT	No	Itra	2	Cured	FK, MMF, steroids
58	Calabro et al	2008	Italy	M,53	19	Legs, papulo-nodule	No	His , Myc	*A.alternata*	DDKT	No	Fluco/ Itra	4	Cured	NA
59	Segner et al	2009	Belgium	M,73	24	Right hand, plaque	No	His, Myc , RT-PCR, ITS	*A. infectoria*	DDKT	Yes	Itra	12	Cured	FK, MMF, steroids
60	Larsen et al	2009	Denmark	M,43	6	Legs, papulo-nodule	No	His, Myc , ITS	*A. alternata*	DDKT	No	Vori	5	Cured	FK, MMF, steroids
61	Vermeire et al	2010	Belgium	F,51	3	Knee, plaque	No	Myc	*A.alternata*	DDKT	Yes	Vori	3	Cured	FK, MMF, steroids
62	Boyce et al	2010	USA	F,45	0	Legs, pustules	No	His , Myc	*Alternaria* spp	HT	Yes	Itra / Cas / Posa	6	Cured	CyA, MMF, steroids
62	Boyce et al	2010	USA	M,62	4	Right hand, papulo-nodule	No	His , Myc	*Alternaria* spp	HT	Yes	Vori	4	Cured	Sirolimus, Aza, steroids
62	Boyce et al	2010	USA	M,51	7	Legs, left thigh and Right ankle, papulo-nodule	No	His , Myc	*Alternaria* spp	CoT	Yes	Itra / Cas	53	DOD	FK, MMF, steroids
62	Boyce et al	2010	USA	F,60	10	Right foot, plaque	No	His , Myc	*Alternaria* spp	CoT	Yes	Itra	4	Cured	FK, MMF, steroids
62	Boyce et al	2010	USA	M,41	17	Legs, papulo-nodule	No	His , Myc	*Alternaria* spp	CoT	Yes	Itra	24	Cured	FK, MMF, steroids

62	Boyce et al	2010	USA	M,36	12	Knee, legs and left forearm, papulo-nodule	No	His , Myc	*Alternaria* spp	DDKT	Yes	Itra	8	Cured	FK, MMF, steroids
62	Boyce et al	2010	USA	M,40	22	Legs, papulo-nodule	No	His , Myc	*Alternaria* spp	CoT	Yes	Itra	3	Cured	FK, MMF, steroids
62	Boyce et al	2010	USA	F,63	36	Right hand, papulo-nodule	No	His , Myc	*Alternaria* spp	PT	Yes	Itra	6	Cured	FK, MMF, steroids
63	Morales et al	2010	Spain	M,63	6	Legs and Arms, ulcerated nodule	No	His , Myc	*Alternaria* spp	HT	No	Cryo/ Itra	6	Cured	FK, MMF, steroids
64	Santiago et al	2010	Portugal	M,55	90	Legs and hand, papulo-nodule	No	NA	*A.alternata*	DDKT	No	Itra/ Amph B / Cryo	4	Cured	FK, steroids
65	Osmond et al	2011	USA	M,57	36	Knee, plaque	No	His , Myc	*Alternaria* spp	HT	Yes	Vori	3	Cured	FK, Aza, steroids
66	Zhang et al	2011	China	M,45	4	legs, papule-nodule	No	His , Myc	*A.infectoria*	KT	No	Terb	12	Cured	FK, mizoribine, steroids
67	Cunha et al	2012	Portugal	M,53	16	Forearm, hands and tibia, papulo-nodule	No	His, Myc , ITS	*A. infectoria*	DDKT	No	Itra	10	Cured	FK, MMF, steroids
68	Seyfarth et al	2012	Germany	M,65	4	Left hand, plaque	No	His, Myc , ITS	*A. infectoria*	DDKT	No	Vori /Cas	3	Cured	FK, MMF, steroids
69	Tambasco et al	2012	Italy	F,64	NA	Legs, plaque	No	His, Myc , ITS	*Alternaria* spp	DDKT	No	Terb	7	Cured	NA
70	Robert et al	2012	France	M,54	8	Legs, papulo-nodule	No	His, Myc , ITS	*A. infectoria*	DDKT	No	Fluco	1	Cured	FK, MMF, steroids
70	Robert et al	2012	France	F,73	60	Right hand, papulo-nodule	Yes	His, Myc , ITS	*A. infectoria*	DDKT	No	Vori	4	Cured - DOD	FK, steroids
70	Robert et al	2012	France	M,56	3	Knee, papulo-nodule	No	His, Myc , ITS	*A. infectoria*	CoT	Yes	Vori/ Posa/ Cas	1	Cured	CyA, MMF, steroids
71	Rammaert et al	2012	France	F,64	30	Right foot, ulcerated nodule	Yes	His, Myc , ITS	*A. infectoria*	HT	No	Itra/ Posa	12	Cured	FK, MMF, steroids
72	Lavergne et al	2012	France	M,63	18	Right elbow, papulo-nodule	No	Myc , molecular identification	*A. alternata*	HT	No	Vori	10	Cured	FK, MMF, steroids
73	Saegeman et al	2012	Belgium	F,52	55	Legs, papulo-nodule	No	His, Myc , ITS	*A. infectoria*	LuT	Yes	Vori	6	DOD	FK, Aza, steroids
74	Severo et al	2012	Brazil	M,27	0,5	Legs and foot, papulo-nodule	No	His, Myc , ITS	*A. alternata*	DDKT	NA	NA	NA	NA	FK
75	Sharikashani et al	2013	Iran	M,37	6	Feet, papulo-nodule	Yes	His , Myc	*Alternaria* spp	HT	No	Vori	3	Cured	CyA, MMF, steroids
76	Ferreira et al	2013	Portugal	M,12	1	Legs, ulcerated nodule	No	His , Myc	*A.alternata*	BMT	No	Posa/ Amph B	1,5	Cured	FK, MMF, steroids
77	Lopes et al	2013	Portugal	M,61	6	Legs, papulo-nodule	No	His, Myc , ITS	*A. infectoria*	DDKT	No	Itra	3	Cured	FK, steroids
77	Lopes et al	2013	Portugal	M,63	14	Legs, papulo-nodule	No	His, Myc , ITS	*A. infectoria*	DDKT	No	Cryo/ Posa	1	Cured	CyA, steroids
77	Lopes et al	2013	Portugal	M,56	120	Right hand, papulo-nodule	No	His , ITS	*A. infectoria*	DDKT	Yes	Itra	3	Cured	FK, MMF, steroids
78	Alhmali et al	2013	Germany	F,65	24	Right wrist and right heel, papulo-nodule	No	NA	*A.infectoria*	LiT	No	CPX/ Fluco	7	Cured	FK, MMF, steroids
79	Secnikova et al	2014	Czech republic	M,60	9	Left arm, ulcerated nodule	Yes	His, Myc , ITS	*A. alternata*	HT	Yes	Vori/ Posa	7	Cured	FK, MMF, steroids
80	Essabbah et al	2014	Tunisia	F,33	9	Foot, papulo-nodule	No	His , Myc	*A.tenuissima*	DDKT	No	Amph B/ Fluco / Cryo	6	Cured	FK, MMF, steroids
81	Michelon et al	2014	Kenia	M,70	50	Hand and feet, papulo-nodule	No	His , Myc	*Alternaria* spp	DDKT	No	Itra	15	Cured	FK, MMF, steroids
82	Coussens et al	2014	Belgium	M,65	6	Legs, ulcerated nodule	No	His , Myc	*A.infectoria*	LiT	No	Fluco	7	Cured	NA
83	Salido-Vallejo et al	2014	Spain	M,64	NA	Foot, papulo-nodule	No	His , Myc	*Alternaria* spp	DDKT	Yes	No	0	Cured	NA
84	Daglar et al	2014	Turkey	M,33	84	Right arm and legs, papulo-nodule	No	His , RT-PCR	*A. infectoria*	DDKT	No	Itra / Amph B	3	Cured	FK, MMF, steroids
85	Gonzalez-Vela et al	2014	Spain	M,60	4	Left hand and legs, papulo-nodule	No	His, Myc , ITS	*A. triticina*	LuT	No	Itra	6	Relapse	FK, MMF, steroids
86	Coutinho et al	2015	Portugal	M,59	9	Legs, papulo-nodule	No	His , Myc	*A.alternata*	DDKT	Yes	Itra/ Cryo	3	Cured	FK, MMF, steroids
86	Coutinho et al	2015	Portugal	F,59	9	Forearm, papulo-nodule	NA	His , Myc	*A.alternata*	DDKT	Yes	Itra/ Cryo	3	Relapse	FK, MMF, steroids
86	Coutinho et al	2015	Portugal	M,59	9	Legs, papulo-nodule	No	His , Myc	*A.alternata*	DDKT	Yes	Itra/ Cryo	3	Cured	FK, MMF, steroids
86	Coutinho et al	2015	Portugal	M,59	9	Legs, papulo-nodule	No	His , Myc	*A.alternata*	DDKT	Yes	Itra/ Cryo	3	Cured	FK, MMF, steroids
86	Coutinho et al	2015	Portugal	M,59	9	Legs, papulo-nodule	No	His , Myc	*A.alternata*	DDKT	Yes	Itra/ Cryo	3	Cured	FK, MMF, steroids
86	Coutinho et al	2015	Portugal	M,59	9	Legs, papulo-nodule	No	His , Myc	*A.alternata*	DDKT	Yes	Itra/ Cryo	3	Cured	FK, MMF, steroids
86	Coutinho et al	2015	Portugal	M,59	9	Legs, papulo-nodule	No	His , Myc	*A.alternata*	DDKT	Yes	Itra/ Cryo	3	Cured	FK, MMF, steroids
87	Demirci et al	2015	Turkey	F,32	4	Knee, ulcerated nodule	No	His , Myc	*Alternaria* spp	DDKT	No	Itra	1	Cured	FK, MMF, steroids
88	Chia-Chi Hsu et al	2015	China	M,61	12	Left lateral ankle, papulo-nodule	No	His , Myc	*Alternaria* spp	DDKT	Yes	Itra/ Vori	3	Cured	FK, MMF, steroids
89	Bras et al	2015	Portugal	M,65	2	Legs and ketf hand, papulo-nodule	No	His, Myc , ITS	*A. alternata, A. infectoria*	LiT	Yes	Itra	3	Cured	FK, MMF, steroids
90	Simpson et al	2016	USA	M,60	NA	Hand, papulo-nodule	No	His , Myc	*Alternaria* spp	HT	Yes	Itra/ Cryo	2	Cured	FK, MMF, steroids
91	Karatas et al	2016	Turkey	M,48	4	Arm, papulo-nodule	No	His, Myc , ITS	*A. alternata*	DDKT	Yes	Itra	12	Cured	FK, MMF, steroids
92	Lyscova et al	2017	Czech republic	M,61	12	Helbow, papulo-nodule	Yes	His, Myc , ITS, partial β-tubulin gene	*A. infectoria*	HT	Yes	Vori/ Posa	7	Cured	FK, MMF, steroids
93	Bajwa et al	2017	USA	M,56	5	Legs, papulo-nodule	No	His , Myc	*Alternaria* spp	PT	No	Posa	18	Cured	FK, MMF, steroids
94	Liu et al	2017	USA	M,66	1,5	Right thumb, papulo-nodule	No	His, Myc , ITS	*A. rosae*	BMT	No	Vori/ Posa	2	DOD	CyA, Sirolimus, MMF, steroids
95	Caviedes et al	2017	Argentina	F,49	6	Knee, plaque	No	His , Myc	*A.alternata*	DDKT	Yes	Itra	3	Cured	FK, MMF, steroids
96	Schuermans et al	2017	Belgium	F,52	NA	Forearm, papulo-nodule	No	His , ITS	*A. infectoria*	LDKT	Yes	Vori/ Itra	NA	Cured	FK, MMF, steroids
97	Salgüero Fernández et al	2018	Spain	M,55	72	Right Arm, papulo-nodule	No	His	*Alternaria* spp	LuT+DDKT	No	Itra/ Clot	1,7	Cured	FK, steroids
98	Dalla Gasperina et al	2019	Italy	M,68	58	Left hand, plaque	No	His, Myc , ITS	*A. alternata*	KT	No	Vori/ Isa/ Posa	1	Cured	FK, MMF, steroids
99	Margheim et al	2019	USA	F,64	10	Diffuse nodules	No	His , Myc	*A.alternata*	HT	Yes	Posa	6	Cured	FK, MMF, steroids
100	Iturrieta-González et al	2019	Spain	M,46	24	Right leg, nodule	No	His , Myc	*Alternaria* spp	LuT	Yes	Vori	0,8	Cured	FK,MMF
101	Campoli et al	2020	Italy	F,56	2	Diffuse ulcerated papules	No	His, Myc , MALDI-TOF MS	*A. alternata*	LiT	Yes	Vori	6	Cured	NA
102	Maisons et al	2022	France	M,69	48	Knee, ulcerated nodule	No	His, Myc , LSU	*A. infectoria*	HT	Yes	Isa/Terb	12	Cured	FK,MMF,steroids
103	Prat-Colilles et al	2025	Spain	F,69	NA	Right leg, plaque-nodule	No	His , Myc	*A.infectoria*	KT	Yes	Amph B/ Vori	2	Cured	FK, MMF, steroids
